# rs2476601 in *PTPN22* gene in rheumatoid arthritis and periodontitis—a possible interface?

**DOI:** 10.1186/s12967-020-02548-w

**Published:** 2020-10-15

**Authors:** Susanne Schulz, Pauline Zimmer, Natalie Pütz, Elisa Jurianz, Hans-Günter Schaller, Stefan Reichert

**Affiliations:** Department of Operative Dentistry and Periodontology, Martin Luther University Halle-Wittenberg, Halle, Germany

**Keywords:** Rheumatoid arthritis, Periodontitis, SNP, *PTPN22*, *PADI4*, *CTLA4*

## Abstract

**Background:**

Rheumatoid arthritis (RA) and periodontitis (PD) are proven to share common risk markers, including genetic factors. In the present study we focused on genetic variants in *PTPN22* (rs2476601), *PADI4 (*rs2240340), *CTLA4* genes (rs3087243) and its impact on RA and PD.

**Materials and methods:**

In the study 111 RA patients and 256 systemically healthy controls were involved. A subdivision of patients and controls was carried out according the severity of periodontitis (no/level 1 PD vs. level 2 PD).

**Results:**

I. Evaluating the genetic impact on the occurrence of RA the T allele of rs2476601 (*PTPN22)* (bivariate: *p* < 0.001; multivariate: *p* = 0.018) and T allele of rs2240340 (*PADI4*) (bivariate: *p* = 0.006; multivariate: *p* = 0.070) were associated with an increased vulnerability to RA.

II. Investigating the genetic influence on level 2 PD the T allele of rs2476601 (*PTPN22)* was shown to be associated with a higher susceptibility to PD within the RA group (bivariate: p = 0.043; multivariate: *p* = 0.024).

III. The T allele of rs2476601 (*PTPN22)* was proven to be a significant marker of RA and level 2 PD comorbidity (bivariate: *p* < 0.001; multivariate: *p* = 0.028).

**Conclusions:**

These results support the thesis that genetic variations may represent a possible link between PD and RA. The study increases knowledge about disease-specific and cross-disease genetic pattern.

## Background

Reciprocal influences between rheumatoid arthritis (RA) and periodontitis (PD) have been strongly emphasized in different studies [1–3]. The bidirectional causal relationship between both chronic destructive inflammatory diseases is remarkably characterized by shared common risk modulators suggesting similar pathogenic pathways [4]. The convincing biological plausibility between both diseases can be, inter alia, attributed to the following mechanisms; effects on microbiological dysbiosis, on inflammatory response and genetic susceptibility.

The impact of genetic risk factors in both RA [5–7] and PD [8, 9] has been intensively studied in recent years. Epidemiological studies revealed that the genetic contribution to rheumatoid arthritis and periodontitis is substantial. About 50% of the risk for development of RA [10] as well as PD [11] has been attributed to genetic factors. Studies indicated a shared genetic association between MHC class II HLA-DBR1 alleles and susceptibility to both RA and PD [4, 12]. In several case control studies, a shared dependence of both diseases on genetic variants in candidate genes, *KCNQ1* [13] and *IFNγ* gene [14] has also been reported.

### Candidate gene approach

These findings suggest a common genetic profile linked to a higher susceptibility to RA and PD. To substantiate this hypothesis, we investigated SNPs in candidate genes that have an important role in pathophysiological mechanisms relevant to both diseases. To this aim, we selected SNPs in three genes that play an important role in the immune response and citrullination, which are key mechanisms in both RA and PD.

Protein tyrosine phosphatase, non-receptor type 22 gene (*PTPN22)* represents a specific lymphophosphatase that plays an important role in T cell signaling due to its inhibiting effect [15, 16]. It was implicated in the etiology of various autoimmune diseases, including RA [15, 17, 18]. Strong evidence of a genetic involvment of *PTPN22* enzyme activity was reported for the first time in 2004 [19]. In detail, a SNP (rs2476601; Arg629Trp) was described, which leads to alteration of the physiological function of *PTPN22* enzyme as negative regulator of T cell activation [19–21]. Furthermore, this genetic variant was shown to be linked with a modification in cytokine profile towards a proinflammatory state [22]. Therefore, a lot of case–control studies were conducted in order to evaluate the potential impact of the SNP rs2476601 in the *PTPN22* gene in the etiology of immune diseases [19–22].

Cytotoxic T-lymphocyte-associated protein 4 gene (*CTLA4)* as a member of the immunoglobulin superfamily is expressed by activated T cells [5, 23]. It was demonstrated to be able to submit an inhibitory signal to T cells suggesting a significant role in the maintenance of immunological homeostasis [23]. A multitude of SNPs were characterized in *CTLA4* gene including rs3087243 located at 3-prime of the end of the *CTLA4* transcript [24].

Protein-arginine deiminase type-4 gene (*PADI4),* one out of 4 human *PADI* isoforms, is assigned an important role in triggering autoimmune diseases especially RA due to its ability to citrullination and its implication in the development of autoantibodies [25, 26]. Furthermore, it could be shown that citrullination occurs also as a physiological process in the gingival epithelium [27]. In the case of severe PD, Engstrom and co-workers proved indeed an increase in citrullination in gingival connective tissue [28]. Genetic variants in *PADI4* have been demonstrated to play a vital role in the etiology of inflammatory diseases [29–32].

Aim of the study:

In order to prove the hypotheses of a common genetic profile associated with a higher prevalence.

1. of RA
2. of PD
3. of RA and PD

we analyzed rs2476601 in *PTPN22*, rs2301888 in *PADI4* and rs3087243 in *CTLA4* gene regarding their significance as periodontal or/and rheumatic risk modulators.

## Materials and methods

### Study population and clinical investigation

In the presented case–control study 367 unrelated subjects of Caucasian origin were included. 111 patients suffered from rheumatoid arthritis (RA) according to current criteria for classifying rheumatoid arthritis [33] and 256 systemically healthy controls without RA were enrolled. All patients examined in the study were classified according to their periodontal status according to the consensus report for “Definition of a periodontitis case and disease progression in risk factor research” [34]. A “level 1 periodontitis” was defined as presence of proximal attachment loss of ≥ 3 mm in ≥ 2 non-adjacent teeth. Controls with vestibular values of clinical attachment loss > 3.5 mm caused by traumatic tooth brushing or orthodontic therapy, CAL according overhanging subgingival restorations or primary endodontic lesions were not considered as cases of periodontitis. Furthermore, pseudo pockets on the last molars with a depth of > 3.5 mm were not considered as periodontitis case [35]. A “level 2 periodontitis” was described as occurrence of proximal attachment loss of ≥ 5 mm in ≥ 30% of teeth present. Taking the new classification system of periodontitis into account the level 2 periodontitis cases included in this study would be categorized as periodontitis stage III or IV, grade B or C [36].

In general, patients and controls were only included if they had a minimum age of 18 years (patients) or 30 years (controls), at least four own teeth, were not pregnant and had not taken antibiotics in the past 3 months or undergone non-surgical periodontal therapy 6 months prior to the examination. The patients and controls had no known medical or general health conditions that might profoundly contribute to development of periodontitis (diabetes mellitus type I or II, Morbus Crohn, coronary heart disease, lupus erythematosus, Behçet disease, oral pemphigus or pemphigoid; except RA in RA-patients). Subjects were not included if they developed gingival overgrowth due to specific drugs such as anti-epileptics, calcium-channel blockers, or cyclosporine.

The periodontal examination of all patients comprised the assessment of plaque index, percentage of sites with bleeding upon probing (BOP), pocket depth (PD: distance between gingiva margin and apical stop of the periodontal probe) and number of missing teeth. To determine the mean clinical attachment loss (CAL: distance between cement-enamel junction and apical stop of the probe) in cases and controls six sites around each tooth were measured and the maximum values were used for the mean value calculations.

The RA free control group comprised of 256 subjects including 161 patients suffering from level 2 periodontitis (presence of proximal attachment loss of ≥ 5 mm in ≥ 30% of teeth present) and 95 patients without or level 1 periodontitis (presence of proximal attachment loss of ≥ 3 mm in ≥ 2 non-adjacent teeth) according to Tonetti and Claffey [34]. This group was recruited consecutively at the Department of Operative Dentistry and Periodontology of the Martin Luther University Halle-Wittenberg from 2005 until 2009. The periodontal examination was carried out by an experienced periodontist using a non-pressure-sensitive periodontal probe (PCPUNC156, Hu-Friedy, Rotterdam, Netherlands).

The RA group (n = 111) included 26 subjects with diagnosed level 2 periodontitis and 85 patients without level 2 periodontitis [34]. RA was diagnosed and treated at the Clinic of Internal Medicine II, Department of Rheumatology, Martin-Luther-University Halle-Wittenberg (Prof. G. Keyßer, Dr. C. Schäfer), at the Department of Rheumatology “Rheumahaus Potsdam” (Dr. M. Bohl-Bühler & Dr. S. Reckert) and at private practices in Magdeburg (Dr. C. Weimann) and Halle (Saale) (Dr. A. Liebhaber; Dr. Th. Linde; Dr. R. Schobeß (Halle). From 2012 until 2016 the RA patients were included consecutively without consideration of periodontal status. For periodontal assessment pressure sensitive periodontal probes (TPS-probe Vivicare, Vivadent, Schaan, Liechtenstein or DB764R Aesculap AG & Co. KG, Tuttlingen, Germany) were used. The dental examiners were instructed and trained in the implementation of both periodontal probes.

### Genetic investigations

Preparation of genomic DNA was carried out by means of QIAamp blood extraction kit (Qiagen, Hilden, Germany) in accordance with the manufacturer’s manual using venous blood.

For the analysis of SNPs specific analyses were established and validated. Genotypes were determined by polymerase chain reaction-restriction fragment length polymorphism (PCR–RFLP) using Mastermix (Promega, Mannheim, Germany). The amplification was performed on the device Eppendorf Mastercycler® gradient (Eppendorf, Hamburg, Germany): PCR-program (2 min 94 °C; 15 cycles: 15 s 92 °C, 30 s 58 °C, 30 s 72 °C; 26 cycles: 30 s 92 °C, 30 s 56 °C, 30 s 72 °C, 5 min 72 °C). The amplified products were digested with restriction enzymes (New England Biolabs, Frankfurt, Germany, according to manufactures protocols) and size-fractionated by electrophoretic separation in 3% agarose gels. The used primers and enzymes are displayed in Table 1.

**Table 1:** Primer and restriction enzymes used for genetic analyses

SNP	PCR primer 5’ 3’		Fragment length	Restriction enzyme
PTPN22: rs2476601	Upper	CTC AAG GCT CAC ACA TCA GC	337bp	RsaI
	Lower	TTG AAT GAA CAA GTG TCA AC		
PADI4: rs2240340	Upper	GCG CAC AAG GAG ATT TCT GAA ATC CCA TTA	131bp	MseI
	Lower	TTG AAT GAA CAA GTG TCA AC		
CTLA4: rs3087243	Upper	CTG GAA ACA AAC TGA AAA ACC	253bp	NlaIII
	Lower	GCC TGT GAT AGT TGA GCT GAG		

### Assessment of *Aggregatibacter actinomycetemcomitans* and *Porphyromonas gingivalis*

Before subgingival scaling was carried out the microbial samples were collected from the deepest pocket of each quadrant (insertion of a sterile paper point for 20 s) and pooled in one tube. Bacterial DNA was isolated applying the QIAamp® DNA Mini Kit (Qiagen, Hilden, Germany) according to the manufacturer’s manual. The subgingival occurrence of *A.a.* and *P.g.* was detected using the micro-Ident® test of HAIN-Diagnostik (Nehren, Germany) according to manufacturer’s protocol. The method was described in detail in [37].

### Statistical analyses

Statistical analyses were carried out using commercially available software (SPSS v.25.0 package, IBM, Chicago, IL). Values of *p* ≤ 0.05 were considered significant. Continuous data were assessed for normal distribution using the Kolmogorov–Smirnov test and the Shapiro–Wilk test. All metric values were not normally distributed. Therefore, data were reported as median, 25/75th-interquartiles. For the statistical evaluation of continuous variables Mann–Whitney U-Test, or Kruskal–Wallis-Test were used. Categorical variables were plotted in contingency tables and evaluated using chi-square test and Yates continuity correction. If the expected cell frequency was < 5, Fisher’s exact test was applied.

Binary logistic regression analysis was used for investigating the impact of polymorphic variants on the occurrence of PD, RA and PD/RA comorbidity considering established confounders.

## Results

### Clinical evaluation

We performed a case–control study in order to evaluate the impact of genetic variants in selected RA-associated candidate genes with the prevalence of RA, PD and RA/PD comorbidity. We focused on the genotype and allele distribution of SNPs rs2476601 (*PTPN22*), rs2240340 (*PADI4*) and rs3087243 (*CTLA4*).

The study group comprised of 367 patients (111 RA patients and 256 systemically healthy controls without RA). The two groups were subdivided according to their periodontal status. The demographical and periodontal characteristics in dependence on RA and PD are displayed in Table 2. In general, patients suffering from RA (n = 111) were significantly older (*p* > 0.001), more often of female gender (*p* = 0.001) and were more often smokers (*p* = 0.003) than controls without RA and level 2 PD (n = 95). RA patients exhibited the more severe dental parameters regarding probing depth (*p* < 0.001) and clinical attachment loss (*p* < 0.001). Furthermore, RA patients frequently complained of tooth loss (*p* < 0.001). Subdividing the group of RA patients according their periodontal status it was obviously that patients suffering from level 2 periodontitis were more often males (*p *= 0.048) and more often smokers (*p* = 0.034). As expected, all RA patients and RA free controls suffering from level 2 periodontitis showed the more level 2 periodontal characteristics.

**Table 2:** Demographical characteristics and periodontal conditions in dependence on rheumatoid arthritis (RA) and periodontitis (PD)

Variable	Control probands (without RA)		RA patients			*p* value**
	No/level 1 PD n = 95	Level 2 PD* N = 161	All n = 111	No/ level 1 PD n = 85	Level 2 PD* n = 26	
Demographical and anamnestic parameters						
Age, years (Median (25–75^th^ IQR))	43.0 (38.0/51.0)	45.0 (38.0/52.0)	56.0 (48.0/63)	56.0 (47.0/63.0)	56.5 (50.0/64.5)	* < 0.001*
Male gender (%)	51.6	37.3	28.8	23.5	46.2	*0.001*
Current smoking (%)	21.1	29.4	23.4	18.8	38.5	*0.003*
Periodontal conditions (Median (25–75th IQR)						
Plaque index (%)	39.0 (28.6/59.3)	57.0 (38.0/82.0)	35.7 (11.5/66.7)	27.0 (7.25/58.55)	64.1 (37.6/84.1)	0.176
Bleeding on probing/tooth (%)	42.0 (23.1/63.0)	82.5 (56.0/100.0)	36.4 (18.2/69.6)	32.0 (16.7/63.25)	58.6 (32.4/89.8)	0.941
Probing depth, mm	2.52 (2.27/2.76)	5.29 (4.52/6.4)	3.0 (2.7/3.7)	2.8 (2.6/3.2)	4.3 (3.78/4.96)	* < 0.001*
Clinical attachment loss, mm	2.8 (2.55/3.14)	6.1 (5.27/7.16)	3.4 (2.9/4.3)	3.1 (2.8/3.7)	5.15 (4.5/6.05)	* < 0.001*
Missing teeth (except 8th)	2.0 (0/4.0)	3.0 (1.0/6.0)	5.0 (2.0/10.0)	4.0 (1.0/10.0)	8.5 (5.0/15.25)	* < 0.001*

*Proximal attachment loss of ≥ 5 mm in ≥ 30% of teeth present

**p value is given for statistical comparison of the control group without RA and no/level 1 PA (n = 95) with the RA total group (n = 111)

Statistical comparisons were made by chi-square test including Yates correction for categorical variables. Continuous variables were analyzed by Mann–Whitney Test and presented as median (25th/75th-interquartiles; IQR, values not normally distributed)

#### Impact of genetic variants on the prevalence on RA

Bivariate comparisons of genetic variants in dependence of RA are displayed in Table 3, multivariate models are shown in Table 4.

**Table 3:** Genotype and allele distribution of SNPs in *PTPN22* (rs2476601), *PADI4* (rs2240340) and *CTLA4* (rs3087243) among RA patients and controls

Variable	Control probands (without RA)		RA patients			*p* value		
	No/ level 1 PD n = 95	Level 2 PD N = 161	All n = 111	No/ level 1 PD n = 85	Level 2 PD n = 26	Prevalence of RA	Prevalence of PD within RA group	Comorbidity PD and RA
	I	II	III	IV	V	I. vs. III	IV. vs. V	I. vs. V
*PTPN22, *rs2476601								
CC genotype (%)	78.9	74.5	60.4	67.1	38.5			
CT genotype (%)	19.0	23.5	36.0	29.4	57.7			
TT genotype (%)	2.1	1.9	3.6	3.5	3.8			
C allele (%)	88.4	86.3	78.4	81.8	67.3			
T allele (%)	11.6	13.7	*21.6↑*	18.2	*32.7↑*	* < 0.001*	*0.043*	* < 0.001*
*PADI4*, rs2240340								
CC genotype (%)	51.6	38.5	36.0	37.6	30.8			
CT genotype (%)	11.6	27.3**↑**	14.5	9.4	*30.7*↑			
TT genotype (%)	36.8	34.2	49.5	52.9	38.5			
C allele (%)	57.4	52.2	43.2	42.4	46.2			
T allele (%)	42.6	47.8	*56.8↑*	57.6	*53.8↑*	*0.006*	0.746	0.200
*CTLA4*, rs3087243								
GG genotype (%)	37.9	33.5	44.1	48.2	30.8			
AG genotype (%)	50.5	47.2	44.2	37.6	65.4			
AA genotype (%)	11.6	19.3	11.7	14.1	3.8↓			
G allele (%)	63.2	57.1	66.2	67.1	63.5			
A allele (%)	36.8	42.9	33.8	32.9	36.5	0.586	0.755	1.000

Statistical comparisons were made by chi-square test including Yates correction for categorical variables

**Table 4:** Binary logistic regression analyses investigating the genetic impact on the prevalence of RA [comparison of the RA total group (n = 111) to the control group without RA and level 2 PD (n = 95)]

Variables	Regression coefficient	Odds ratio	95% confidence interval		*p* value
			Lower	Upper	
a) T allele of rs2476601 in *PTPN22*					
Age	0.064	1.066	1.046	1.087	< 0.001
Male gender	− 0.987	0.373	0.232	0.599	< 0.001
Current smoker	0.712	2.041	1.276	3.257	0.003
*P.g.* positive	1.133	3.106	1.799	5.405	< 0.001
*A.a.* positive	− 1.747	0.174	0.076	0.402	< 0.001
T allele	*0.779*	*2.179*	*1.143*	*4.149*	*0.018*

b) T allele of rs2240340 in *PADI4*					
Age	0.063	1.065	1.045	1.086	< 0.001
Male gender	− 0.968	0.380	0.237	0.610	< 0.001
Current smoker	0.780	2.183	1.364	3.484	0.001
*P.g.* positive	1.082	2.950	1.695	5.128	< 0.001
*A.a.* positive	− 1.697	0.183	0.079	0.422	< 0.001
T allele	0.425	1.529	0.965	2.42	0.070
T allele	*0.493*	*1.637*	*1.013*	*2.645*	*0.044*

Age, gender, smoking status, and the occurrence of *Porphyromonas gingivalis* (*P.g.*) and Agregatibacter actinomycetemcomitans (*A.a*) were considered as confounding factors

The SNP rs2476601 in *PTPN22* gene was associated with a higher prevalence of RA in our study (Table 3). RA patients were significantly more frequent carriers of the T allele carriers than RA free controls without or level 1 periodontitis (*p* < 0.001). Considering age, gender, smoking status and the subgingival occurrence of *A.a.* and *P.g.* as confounding factors in binary logistic regression analyses the genetic impact of T allele on RA could be proven (*p* = 0.018) (Table 4). Considering only RA patients and RA free controls both suffering from level 2 periodontitis the effect of the T allele (bivariate: *p* < 0.001; multivariate: *p* = 0.032) on the prevalence of RA could be confirmed.

Investigating the importance of rs2240340 in *PADI4* gene the T allele was proven to be associated with a higher susceptibility to RA comparing the total RA group to RA free controls without or level 1 periodontitis (*p* = 0.006). In multivariate risk model (binary logistic regression analyses), including age, gender, smoking status, and the occurrence of *A.a.* and *P.g.* as confounding factors, the T allele (*p* = 0.070, Table 4) could not be indicated as independently associated with RA (Table 4). Increasing age, female gender, smoking and the occurrence of *P.g.* and the absence of *A.a.*, however, could be shown as significant risk factors for RA in this complex risk model (Table 4). Furthermore, the T allele of rs2240340 in *PADI4* gene was associated with increased risk of RA comparing RA patients and RA free controls without or level 1 PD (bivariate: p = 0.006; multivariate: *p* = 0.044).

The rs3087243 in *CTLA4* gene was not confirmed to be in association with rheumatoid arthritis in bivariate and multivariate risk models.

#### Effects of genetic variants on the prevalence of level 2 PD

In a subsequent evaluation, possible associations between genetic variants in *PTPN22*, *PADI4*, and *CTLA4* gene and the prevalence of level 2 PD were investigated.

Within the RA group the T allele of rs2476601 in *PTPN22* gene was associated with the occurrence of level 2 PD (bivariate: *p* = 0.043; multivariate: *p* = 0.024) (Table 3, Table 5).

**Table 5:** Binary logistic regression analyses investigating the genetic impact of T allele of rs2476601 in *PTPN22* on level 2 PD [comparison within the RA group: RA patients without level 2 PD (n = 85) to RA patients with level 2 PD (n = 26)]

Variables	Regression coefficient	Odds ratio	95% confidence interval		*p* value
			Lower	Upper	
T allele of rs2476601 in PTPN22					
Age	0.020	1.020	0.992	1.049	0.162
Male gender	0.990	2.691	1.331	5.441	0.006
Current smoker	1.031	2.725	1.316	5.988	0.008
*P.g.* positive	1.132	3.106	1.529	6.289	0.002
*A.a.* positive	− 1.067	0.344	0.067	17.73	0.202
T allele	*0.901*	*2.463*	*1.126*	*5.376*	*0.024*

Age, gender, smoking status, and the occurrence of *Porphyromonas gingivalis* (*P.g.*) and Agregatibacter actinomycetemcomitans (*A.a*) were considered as confounding factors

Regarding the allele distribution of rs2240340 in *PADI4* gene as well as the rs3087243 in *CTLA4* gene no association to PD could be shown.

#### Importance of genetic variants on PD and RA as comorbidities

Patients suffering from level 2 PD and RA simultaneously were more frequently T allele carriers of rs2476601 (*PTPN22* gene) than patients without/level 1 PD or RA (*p* < 0.001; Table 3). In multivariate risk model the T allele had predictive value for level 2 PD and RA (*p* = 0.028, Table 6).

**Table 6:** Binary logistic regression analyses investigating the genetic impact of T allele of rs2476601 in *PTPN22* on both diseases (level 2 PD and RA) [comparison of controls without RA or level 2 PD (n = 95) vs. patients suffering from both, RA and level 2 PD (n = 26)]

Variables	Regression coefficient	Odds ratio	95% confidence interval		*p* value
			Lower	Upper	
T allele of rs2476601 in PTPN22					
Age	0.116	1.123	1.076	1.173	< 0.001
Male gender	0.008	1.008	0.423	2.402	0.986
Current smoker	1.868	6.493	2.481	16.95	< 0.001
*P.g.* positive	2.286	9.804	3.846	1.04	< 0.001
*A.a.* positive	− 2.831	0.059	0.009	0.368	0.002
T allele	*1.136*	*3.115*	*1.134*	*8.547*	*0.028*

According to rs2240340 in *PADI4* gene and rs3087243 in *CTLA4* gene no differences in the distribution of alleles were obvious.

## Discussion

### Clinical evaluation

In the present association study the patient group comprised of RA patients suffering from periodontitis of varying severity (n = 111). A second group of patients exhibit no signs of RA but patients were affected by level 2 periodontitis (n = 161). In the control group systemically healthy patients without/level 1 PD were included (n = 95). In accordance the overall risk pattern of RA, the RA patients were significantly older than controls (Table 2, *p* < 0.001) [38]. Furthermore, it is generally accepted that women, mainly in their 4th or 5th decade, are most frequently affected by RA [39]. This correlation was also confirmed in our study (Table 2, *p* = 0.001). Smoking, another major and acknowledged risk factor for RA [40], was also confirmed in the present study (Table 2, *p* = 0.003). In accordance with various studies RA patients exhibited the more severe periodontal symptoms including probing depth (Table 2, *p* < 0.001), clinical attachment loss (Table 2, *p* < 0.001) and were more frequently affected by tooth loss (Table 2, *p* < 0.001) [41, 42].

Investigating the influence of demographic and clinical parameters on the occurrence of level 2 PD the established periodontal risk pattern regarding gender and smoking status was confirmed [43]. Within the RA group patients suffering from level 2 PD were more often males (*p* = 0.048) and smokers (*p* = 0.034).

#### Impact of genetic variants on the prevalence on RA

In the present study two SNPs, namely rs2476601 in the *PTPN22* and rs2240340 in *PADI4* genes, were associated with the occurrence of RA.

##### PTPN22

Patients presenting the T allele of rs2476601 in *PTPN22* gene were significant more often affected by RA regardless of their periodontal status (Table 3). In different case–control studies the influence of this genetic variant on the etiology of RA was discussed controversially [44, 45]. However, the results presented here are in accordance with a large scale meta-analysis where the T allele [6, 20] was associated with RA susceptibility. A stratification of subjects according to their ethnic background confirmed that *PTPN22* T allele was significantly associated with RA in European patients [20]. Furthermore, the present study was able to provide evidence that even when other risk factors of RA were included in multivariate regression models, the T allele represent an independent risk modulator for RA. In the multivariate analyses increasing age, female gender, smoking,the subgingival occurrence of *P.g.* and the absence of *A.a.* were also proven to be independent risk factors of RA. This is consistent with already accepted findings regarding the established risk factors of RA [46, 47]. The only exception in this context is the absence of *A.a.* as a risk factor for RA in this multivariate evaluation. This is striking since *A.a.* was shown to be involved in citrullination and increased formation of anti-CCP due to affecting human citrullinating enzymes [48, 49].

##### PADI4

Peptidyl arginine deiminase type 4 polymorphisms have been demonstrated to play a vital role in the etiology of RA [32]. Nevertheless, the results were inconclusive [30, 32, 50, 51]. A lot of meta-analyses could approve an association of this SNP with Asian populations but not Caucasians [32, 49, 50]. In contrast, Lee et al. (2007) proved in their meta-analysis a significantly increased risk for TT genotype carriers to suffer from RA in European populations [30]. In line with this result, we could confirm the T allele as a significant predictor for occurrence of RA evaluating RA patients regardless of the periodontal status as well when considering only RA patients without/level 1 PD (Table 3). In multivariate analysis the T allele was only to proven to be an independent risk marker in RA patients without/level 1 PD (Table 4). For the total RA group not the T allele but other RA risk factors, including increasing age, female gender, smoking status, occurrence of *P.g.* as well as the absence of *A.a.* were shown to be indicators for RA in the present study.

##### CTLA4

The GG genotype of this SNP was shown to represent a risk modulator for autoimmune diseases possibly due to the G allele dependent transcriptional downregulation of *CTLA4* [24]. A significant association between the G allele and the occurrence of RA was confirmed in a large-scale meta-analysis in European population [6]. However, the data available are not consistent in this respect. The inconsistent results could be explained, inter alia, by a different stratification of the patient groups. In the study by Luterek-Puszyńska and co-workers no association of SNP rs3087243 in *CTLA4* gene and RA in the Polish population was proven [52]. In line with this result, we were also unsuccessful in our study in confirming the influence of SNPs rs3087243 in *CTLA4* gene on the occurrence of RA. However, a very slight increase in G allele carriers was observed among RA patients (Table 3).

#### Effects of genetic variants on the prevalence of level 2 PD

The occurrence of PD was associated with allele distribution of rs2476601 in the *PTPN22* gene but not with allelic constellation of rs2240340 in *PADI4* and rs3087243 in *CLTA4* genes.

##### PTPN22

For the first time in the present study, an association between SNP rs2476601 in the *PTPN22* gene and the occurrence of level 2 PD was examined. A significant association was shown between the presence of the T allele and level 2 PD in patients with RA (Tables 3, 5). In multivariate analyses the T allele was confirmed as an independent periodontal risk factor.

In accordance with the established view on the aetiology of periodontitis the host response is recognized as the main cause of periodontal tissue damage. The involvement of immune cells, including T cell populations has been clearly demonstrated [53]. *PTPN22* is among the molecules involved in T cell regulation. Taking into account the functional effects of this SNP as negative regulator of T cell activation [19] as well as a potential modulator of the cytokine profile [22] an involvement of this SNP in the aetiology of PD could be explainable. Although some studies have found an association of *PTPN22* to inflammatory diseases, there have been no trials on severe periodontitis to date [15, 17, 18].

##### PADI4

The present study was the earliest to investigate possible associations between SNP rs2240340 in *PADI4* gene and level 2 periodontitis. But no association considering the allele constellation could be shown in this study. It is clearly established that the initial reaction in periodontitis is the local host response to periodontal pathogens in the periodontal pocket. In this context, the recruitment of inflammatory cells is associated with increased apoptosis and necrosis of neutrophils in gingival connective tissue, which may lead to deamination of arginine residues and hypercitrullination of proteins [54]. And indeed, hypercitrullination accompanied with an increase in *PADI4* expression at mRNA and protein level was proven in gingival connective tissue in the case of severe periodontitis [28].

##### CTLA4

In the context of periodontitis, the immune response must be controlled very sensitively in order to effectively prevent the spread of pathogenic microorganisms and prevent collateral damage to the tissue, respectively. For the maintenance of immune homeostasis a regulated immuno-inflammatory response, including the presence of immune regulatory cells, is essential [55]. It could be shown that there was a higher frequency of immune regulatory cells expressing *CTLA4* in periodontal lesions than in gingivitis confirmed by changes in *CTLA4* expression [56, 57]. Furthermore, *CTLA4* was supposed to be involved in suppression of osteoclast differentiation and activation an important advance in the etiology of periodontitis [53]. These results support the hypothesis that *CTLA4* may also play an important role in the pathogenesis of periodontal disease.

Since rs3087243 in *CLTA4* gene was shown to affect transcriptional regulation of *CTLA4* gene [24] associated with increased bone destruction [58, 59] one could expect the implication of this SNP in level 2 PD.

#### Importance of genetic variants on PD and RA as comorbidities

In this study, candidate gene approach was used to identify common genetic interfaces between RA and PD. Here we could confirm associations between rs2476601 in *PTPN22* gene but not rs2240340 in *PADI4* gene and rs3087243 in *CTLA4* gene and comorbidity of RA and PD.

##### PTPN22

Because of its functional consequences on *PTPN22* enzyme activity [19] as well as on cytokine expression [22] SNP rs2476601 was supposed to be involved in the etiology of inflammatory diseases including RA and PD. We could confirm for the first time the T allele of SNP rs2476601 in *PTPN22* gene as independent risk factor for both, RA and PD (Table 3, Table 6). *PTPN22* and its functional important genetic variant rs2476601 could therefore provide a biological plausible link between both inflammatory diseases.

### Study limitations

A case–control approach was chosen for the present study. It was designed to provide hypotheses on possible links between genetic variants and level 2 PD or RA or comorbidity. However, in view of the study design, it is not feasible to verify these hypotheses.

Moreover, patients with rheumatoid arthritis may be restricted in their oral hygiene during acute phases of the disease. These restrictions could trigger the development or progression of periodontal disease in high risk patients.

One of the strengths of the study was the homogeneous ethnicity and the strict inclusion and exclusion criteria of the study participants. Due to this, the sample size was relatively small. This may lead to a potential bias, as the probability of a type II error will increase, distorting the results. It would therefore be beneficial to replicate the study results in a further study. A further limitation of the study is the disproportionate size of the study groups, which could influence reliability. Moreover, the persons involved in the study are not necessarily representatives of the total population. The data obtained should be interpreted with caution since they can be considered applicable for Caucasian individuals of central Germany only. Extrapolation to the general population is not rational.

## Conclusions

The present results support the hypothesis that SNP rs2476601 of the *PTPN22* gene, involved in T- and B-cell signalling, might represent an independent shared genetic risk factor for PD and RA. This would strengthen the thesis that there could be a shared genetic interface for both inflammatory diseases.

## Data Availability

The datasets used and/or analysed during the current study are available from the corresponding author on reasonable request.

## Abbreviations

A.a.: Aggregatibacter actinomycetemcomitans
*CTLA4*: Cytotoxic T-lymphocyte-associated protein 4
IFNγ: Nterferon gamma
*PADI4*: Protein-arginine deiminase type-4
PD: Periodontitis
*PTPN22*: Protein tyrosine phosphatase, non-receptor type 22
P.g.: Porphyromonas gingivalis
RA: Rheumatoid arthritis
SNP: Small nuclear polymorphism
vs.: Versus

## References

[1] Tang Q, Fu H, Qin B, Hu Z, Liu Y, Liang Y (2017). Possible link between rheumatoid arthritis and periodontitis: a systematic review and meta-analysis. Int J Periodont Rest.

[2] de Smit M, Westra J, Brouwer E, Janssen KMJ, Vissink A, van Winkelhoff AJ (2015). Periodontitis and rheumatoid arthritis: what do we know?. J Periodontol.

[3] de Molon RS, Rossa C, Thurlings RM, Cirelli JA, Koenders MI (2019). Linkage of periodontitis and rheumatoid arthritis: current evidence and potential biological interactions. Int J Mol Sci.

[4] Ceccarelli F, Saccucci M, Di Carlo G, Lucchetti R, Pilloni A, Pranno N (2019). Periodontitis and rheumatoid arthritis: the same inflammatory mediators?. Mediat Inflamm..

[5] Zamanpoor M (2019). The genetic pathogenesis, diagnosis and therapeutic insight of rheumatoid arthritis. Clin Genet.

[6] Zhang W, Xu P, Chen Z, Cheng Y, Li X, Mao Q (2018). IL-13 -1112 polymorphism and periodontitis susceptibility: a meta-analysis. BMC Oral Health.

[7] Okada Y, Wu D, Trynka G, Raj T, Terao C, Ikari K (2014). Genetics of rheumatoid arthritis contributes to biology and drug discovery. Nature.

[8] Ding J, Orozco G (2019). Identification of rheumatoid arthritis causal genes using functional genomics. Scand J Immunol.

[9] Munz M, Richter GM, Loos BG, Jepsen S, Divaris K, Offenbacher S (2019). Meta-analysis of genome-wide association studies of aggressive and chronic periodontitis identifies two novel risk loci. Eur J Hum Genet.

[10] Scott DL, Wolfe F, Huizinga TWJ (2010). Rheumatoid arthritis. Lancet.

[11] Michalowicz BS, Diehl SR, Gunsolley JC, Sparks BS, Brooks CN, Koertge TE (2000). Evidence of a substantial genetic basis for risk of adult periodontitis. J Periodontol.

[12] Koziel J, Mydel P, Potempa J (2014). The link between periodontal disease and rheumatoid arthritis: an updated review. Curr Rheumatol Rep.

[13] Kobayashi T, Kido JI, Ishihara Y, Omori K, Ito S, Matsuura T (2018). The KCNQ1 gene polymorphism as a shared genetic risk for rheumatoid arthritis and chronic periodontitis in Japanese adults: a pilot case-control study. J Periodontol.

[14] Schulz S, Pütz N, Jurianz E, Schaller HG, Reichert S (2019). Are there any common genetic risk markers for rheumatoid arthritis and periodontal diseases? A case-control study. Mediat Inflamm.

[15] Klareskog L, Catrina AI, Paget S (2009). Rheumatoid arthritis. Lancet.

[16] Klareskog L, Rönnelid J, Saevarsdottir S, Padyukov L, Alfredsson L (2020). The importance of differences; on environment and its interactions with genes, and immunity in the causation of rheumatoid arthritis. J Intern Med.

[17] Brownlie RJ, Zamoyska R, Sallmond RJ (2018). Regulation of autoimmune and anti-tumour T-cell responses by PTPN22. Immunology.

[18] Bottini N, Vang T, Cucca F, Mustelin T (2006). Role of *PTPN22* in type 1 diabetes and other autoimmune diseases. Semin Immunol.

[19] Begovich AB, Carlton VE, Honigberg LA, Schrodi SJ, Chokkalingam AP, Alexander HC (2004). A missense single-nucleotide polymorphism in a gene encoding a protein tyrosine phosphatase (*PTPN22*) is associated with rheumatoid arthritis. Am J Hum Genet.

[20] Elshazli R, Settin A (2015). Association of *PTPN22* rs2476601 and STAT4 rs7574865 polymorphisms with rheumatoid arthritis: a meta-analysis update. Immunobiology.

[21] Bottini N, Musumeci L, Alonso A, Rahmouni S, Nika K, Rostamkhani M (2004). A functional variant of lymphoid tyrosine phosphatase is associated with type I diabetes. Nat Genet.

[22] Ghorban K, Ezzeddini R, Eslami M, Yousefi B, Sadighi Moghaddam B, Tahoori MT (2019). PTPN22 1858 C/T polymorphism is associated with alteration of cytokine profiles as a potential pathogenic mechanism in rheumatoid arthritis. Immunol Lett.

[23] Magistrelli G, Jeannin P, Herbault N, De Benoit Coignac A, Gauchat JF, Bonnefoy JY (1999). A soluble form of CTLA-4 generated by alternative splicing is expressed by nonstimulated human T cells. Eur J Immunol.

[24] Ueda H, Howson JM, Esposito L, Heward J, Snook H, Chamberlain G (2003). Association of the T-cell regulatory gene CTLA4 with susceptibility to autoimmune disease. Nature.

[25] Yamada R, Suzuki A, Chang X, Yamamoto K (2003). Peptidylarginine deiminase type 4: identification of a rheumatoid arthritis-susceptible gene. Trends Mol Med.

[26] Bax M, van Heemst J, Huizinga TW, Toes RE (2011). Genetics of rheumatoid arthritis: what have we learned?. Immunogenetics.

[27] Nesse W, Westra J, van der Wal JE, Abbas F, Nicholas AP, Vissink A (2012). The periodontium of periodontitis patients contains citrullinated proteins which may play a role in ACPA (anti-citrullinated protein antibody) formation. J Clin Periodontol.

[28] Engstrom M, Eriksson K, Lee L, Hermansson M, Johansson A, Nicholas AP (2018). Increased citrullination and expression of peptidylarginine deiminases independently of *P. gingivalis* and *A. actinomycetemcomitans* in gingival tissue of patients with periodontitis. J Transl Med.

[29] Chen CC, Isomoto H, Narumi Y, Sato K, Oishi Y, Kobayashi T (2008). Haplotypes of PADI4 susceptible to rheumatoid arthritis are also associated with ulcerative colitis in the Japanese population. J Clin Immunol.

[30] Lee YH, Rho YH, Choi SJ, Ji JD, Song GG (2007). PADI4 polymorphisms and rheumatoid arthritis susceptibility: a meta-analysis. Rheumatol Int.

[31] Massarenti L, Enevold C, Damgaard D, Ødum N, Nielsen CH, Jacobsen S (2019). Peptidylarginine deiminase-4 gene polymorphisms are associated with systemic lupus erythematosus and lupus nephritis. Scand J Rheumatol.

[32] Lee YH, Bae SC (2016). Association between susceptibility to rheumatoid arthritis and PADI4 polymorphisms: a meta-analysis. Clin Rheumatol.

[33] Aletaha D, Neogi T, Silman AJ, Funovits J, Felson DT, Bingham CO (2010). Rheumatoid arthritis classification criteria an american college of rheumatology/European league against rheumatism collaborative initiative. Arthritis Rheum.

[34] Tonetti MS, Claffey N, European Workshop in Periodontology group C (2005). Advances in the progression of periodontitis and proposal of definitions of a periodontitis case and disease progression for use in risk factor research. J Clin Periodontol.

[35] Reichert S, Schlumberger W, Dähnrich C, Hornig N, Altermann W, Schaller HG (2015). Association of levels of antibodies against citrullinated cyclic peptides and citrullinated α-enolase in chronic and aggressive periodontitis as a risk factor of Rheumatoid arthritis: a case control study. J Transl Med.

[36] Caton J, Armitage G, Berglundh T, Chapple ILC, Jepsen S, Kornman KS (2018). A new classification scheme for periodontal and peri-implant diseases and conditions—introduction and key changes from the 1999 classification. J Clin Periodontol.

[37] Schulz S, Zissler N, Altermann W, Klapproth J, Zimmermann U, Gläser C (2008). Impact of genetic variants of CD14 and TLR4 on subgingival periodontopathogens. Int J Immunogenet.

[38] Agnihotri R, Gaur S (2014). Rheumatoid arthritis in the elderly and its relationship with periodontitis: a review. Geriatr Gerontol Int.

[39] Austad C, Kvien TK, Olsen IC, Uhlig T (2015). Health status has improved more in women than in men with rheumatoid arthritis from 1994 to 2009: results from the Oslo rheumatoid arthritis register. Ann Rheum Dis.

[40] Källberg H, Ding B, Padyukov L, Bengtsson C, Rönnelid J, Klareskog L (2011). Smoking is a major preventable risk factor for rheumatoid arthritis: estimations of risks after various exposures to cigarette smoke. Ann Rheum Dis.

[41] Rodríguez-Lozano B, González-Febles J, Garnier-Rodríguez JL, Dadlani S, Bustabad-Reyes S, Sanz M (2019). Association between severity of periodontitis and clinical activity in rheumatoid arthritis patients: a case-control study. Arthritis Res Ther.

[42] Kim JW, Park JB, Yim HW, Lee J, Kwok SK, Ju JH (2019). Rheumatoid arthritis is associated with early tooth loss: results from korea national health and nutrition examination survey V to VI. Korean J Intern Med.

[43] Bouchard P, Carra MC, Boillot A, Mora F, Rangé H (2017). Risk factors in periodontology: a conceptual framework. J Clin Periodontol.

[44] Abbasi Z, Kazemi Nezhad SR, Pourmahdi-Broojeni M, Rajaei E (2017). Association of PTPN22 rs2476601 polymorphism with rheumatoid arthritis and celiac disease in Khuzestan Province, Southwestern Iran. Iran Biomed J.

[45] El-Lebedy D, Raslan H, Ibrahim A, Ashmawy I, El-Aziz SA, Mohammed AM (2017). Association of STAT4 rs7574865 and PTPN22 rs2476601 polymorphisms with rheumatoid arthritis and non-systemically reacting antibodies in Egyptian patients. Clin Rheumatol.

[46] Deane KD, Demoruelle MK, Kelmenson LB, Kuhn KA, Norris JM, Holers VM (2017). Genetic and environmental risk factors for rheumatoid arthritis. Best Pract Res Cl Rh.

[47] Ebbers M, Lübcke PM, Volzke J, Kriebel K, Hieke C, Engelmann R (2018). Interplay between *P. gingivalis, F. nucleatum* and *A. actinomycetemcomitans *in murine alveolar bone loss, arthritis onset and progression. Sci Rep.

[48] Reichert S, Jurianz E, Natalie P, Schlumberger W, Dähnrich C, Johannsen N (2020). Is periodontitis a prognostic factor in order to indicate antibodies against citrullinated peptides in patients with rheumatoid arthritis?. Clin Exp Rheumatol.

[49] König MF, Abusleme L, Reinholdt J, Palmer RJ, Teles RP, Sampson K (2016). Aggregatibacter actinomycetemcomitans-induced hypercitrullination links periodontal infection to autoimmunity in rheumatoid arthritis. Sci Transl Med.

[50] Hou S, Gao GP, Zhang XJ, Sun L, Peng WJ, Wang HF (2013). PADI4 polymorphisms and susceptibility to rheumatoid arthritis: a meta-analysis. Mod Rheumatol.

[51] Lu C, Xu K, Guo H, Peng K, Yang Z, Hao YQ (2018). The relationship of PADI4_94 polymorphisms with the morbidity of rheumatoid arthritis in Caucasian and Asian populations: a meta-analysis and system review. Clin Rheumatol.

[52] Luterek-Puszyńska K, Malinowski D, Paradowska-Gorycka A, Safranow K, Pawlik A (2017). CD28, CTLA-4 and CCL5 gene polymorphisms in patients with rheumatoid arthritis. Clin Rheumatol.

[53] Meyle J, Chapple I (2000). Molecular aspects of the pathogenesis of periodontitis. Periodontology.

[54] Potempa J, Mydel P, Koziel J (2017). The case for periodontitis in the pathogenesis of rheumatoid arthritis. Nat Rev Rheumatol.

[55] Garlet GP, Sfeir CS, Little SR (2014). Restoring host-microbe homeostasis via selective chemoattraction of Tregs. J Dent Res.

[56] Nakajima T, Ueki-Maruyama K, Oda T, Ohsawa Y, Ito H, Seymour GJ (2005). Regulatory T-cells infiltrate periodontal disease tissues. J Dent Res.

[57] Cardoso CR, Garlet GP, Moreira AP, Junior WM, Rossi MA, Silva JS (2008). Characterization of CD4+CD25+ natural regulatory T cells in the inflammatory infiltrate of human chronic periodontitis. J Leukoc Biol.

[58] Bozec A, Zaiss MM (2017). T regulatory cells in bone remodelling. Curr Osteoporos Rep.

[59] Alvarez C, Rojas C, Rojas L, Cafferata EA, Monasterio G, Vernal R (2018). Regulatory T lymphocytes in periodontitis: a translational view. Mediat Inflamm.

